# Discrete Dynamics Model for the Speract-Activated Ca^2+^ Signaling Network Relevant to Sperm Motility

**DOI:** 10.1371/journal.pone.0022619

**Published:** 2011-08-16

**Authors:** Jesús Espinal, Maximino Aldana, Adán Guerrero, Christopher Wood, Alberto Darszon, Gustavo Martínez-Mekler

**Affiliations:** 1 Instituto de Ciencias Fsicas, Universidad Nacional Autónoma de México, Cuernavaca, Morelos, México; 2 Centro de Ciencias de la Complejidad, Ciudad Universitaria, México, México; 3 Departamento de Genética del Desarrollo y Fisiologa Molecular, Instituto de Biotecnologa, Universidad Nacional Autónoma de México, Cuernavaca, Morelos, México; 4 Centro Internacional de Ciencias, Cuernavaca, Morelos, México; University of Illinois at Urbana-Champaign, United States of America

## Abstract

Understanding how spermatozoa approach the egg is a central biological issue. Recently a considerable amount of experimental evidence has accumulated on the relation between oscillations in intracellular calcium ion concentration ([Ca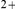]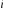) in the sea urchin sperm flagellum, triggered by peptides secreted from the egg, and sperm motility. Determination of the structure and dynamics of the signaling pathway leading to these oscillations is a fundamental problem. However, a biochemically based formulation for the comprehension of the molecular mechanisms operating in the axoneme as a response to external stimulus is still lacking. Based on experiments on the *S. purpuratus* sea urchin spermatozoa, we propose a signaling network model where nodes are discrete variables corresponding to the pathway elements and the signal transmission takes place at discrete time intervals according to logical rules. The validity of this model is corroborated by reproducing previous empirically determined signaling features. Prompted by the model predictions we performed experiments which identified novel characteristics of the signaling pathway. We uncovered the role of a high voltage-activated 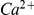 channel as a regulator of the delay in the onset of fluctuations after activation of the signaling cascade. This delay time has recently been shown to be an important regulatory factor for sea urchin sperm reorientation. Another finding is the participation of a voltage-dependent calcium-activated 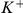 channel in the determination of the period of the 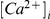 fluctuations. Furthermore, by analyzing the spread of network perturbations we find that it operates in a dynamically critical regime. Our work demonstrates that a coarse-grained approach to the dynamics of the signaling pathway is capable of revealing regulatory sperm navigation elements and provides insight, in terms of criticality, on the concurrence of the high robustness and adaptability that the reproduction processes are predicted to have developed throughout evolution.

## Introduction

Fertilization requires communication between mature and competent male and female gametes so that they may fuse. For this process, a proper understanding of sperm navigation towards the egg is essential. Sperm swimming is dictated in nearly all organisms by the behavior of its flagellum which presents a highly conserved internal structure [1]. In many species the female gametes secrete chemoattractants to guide homologous sperm towards their source. In sea urchins, the eggs are surrounded by a jelly layer that contains sperm-activating peptides (SAPs) that diffuse and bind to receptors on the sperm flagella. This triggers a signaling pathway leading to an intracellular 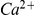 concentration (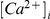) response, which consists in a sustained (tonic) increase in 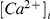 with superimposed fluctuations on that response (supratonic or phasic) [2]. SAPs have been isolated from the egg investments of a variety of sea urchin species and are known to modulate the motility of their homologous spermatozoa [3].

The first characterized and most widely studied member of the SAP family is speract, a decapeptide isolated from *Strongylocentrotus purpuratus* sea urchin eggs [3], [4]. Current models propose that after speract binds to its receptor in the sperm flagella, a membrane guanylate cyclase is activated, increasing the levels of cyclic GMP (cGMP), which leads to the opening of cGMP-regulated 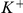 channels that hyperpolarize spermatozoa [5]–[8]. Evidence indicates that the hyperpolarization removes inactivation of voltage-gated 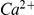 channels, which subsequently open causing a depolarization [7], [9]. It has been suggested that the alternation between hyperpolarization and depolarization in the membrane potential drives the 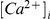 oscillations [2], [7], [10], [11]. The fast transient increase in these flagellar oscillations of the 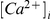 has been associated with the transient modifications in flagellar curvature that prompt sea urchin spermatozoa to undergo a sharp turning event [2], [12]–[14]. Since these turning events are an essential component of sperm motility and reorientation, it is important to understand the molecular mechanisms that generate them.

Several phenomenological models based on mechanical and hydrodynamic considerations have been proposed to describe the motion of the spermatozoon [15]–[20]. These models have successfully reproduced how the form and beating characteristics of the flagellum determine the swimming direction. However, it is still unknown how the 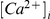 and the underlying signaling network controlling it, shape the bending of the flagellum. In order to fully understand the motility of the spermatozoon, it is necessary to go beyond phenomenological models and base this motility on molecular grounds. As a first step in that direction, here we construct a model of the signaling network that could regulate the levels of 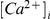 in the flagellum of the sea urchin spermatozoon and analyze its dynamical properties.

In similar prior studies, a set of coupled differential equations has often been defined for this type of analysis [21], [22]. This involves the knowledge of many reaction constants that are frequently difficult to determine and generally requires numerical solutions for a nonlinear problem. However, recent work shows that simpler models, based on the regulatory logic of the interactions rather than on their kinetic details, capture essential aspects of the regulation dynamics and are able to reproduce experimental observations [23]. Therefore, here we adopt a discrete dynamics formalism acting on a logic representation for the pathway that has previously been employed for analysis of genetic regulatory networks [21]. Elements of the pathway are considered as nodes and interactions correspond to links. In this approach, the calculations are easily implemented, flexible, computationally efficient and meaningful. This can be appreciated in the applet we have developed mentioned further on in the text. In addition, general qualitative features and logical relations amongst system components can be readily studied. In our work the incorporation of experimental findings in the construction of the evolution rules strongly contributes to the reliability of the model. Within the framework we have adopted, we are able to determine previously undetected regulatory mechanisms for the temporal behavior of 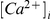, and have for the first time established that the signaling pathway presents an interesting property frequently found in living systems [24]–[26], where robustness and adaptability are concurrent with the highest probability [27].

## Methods

### The Signaling Pathway

**Figure 1:**
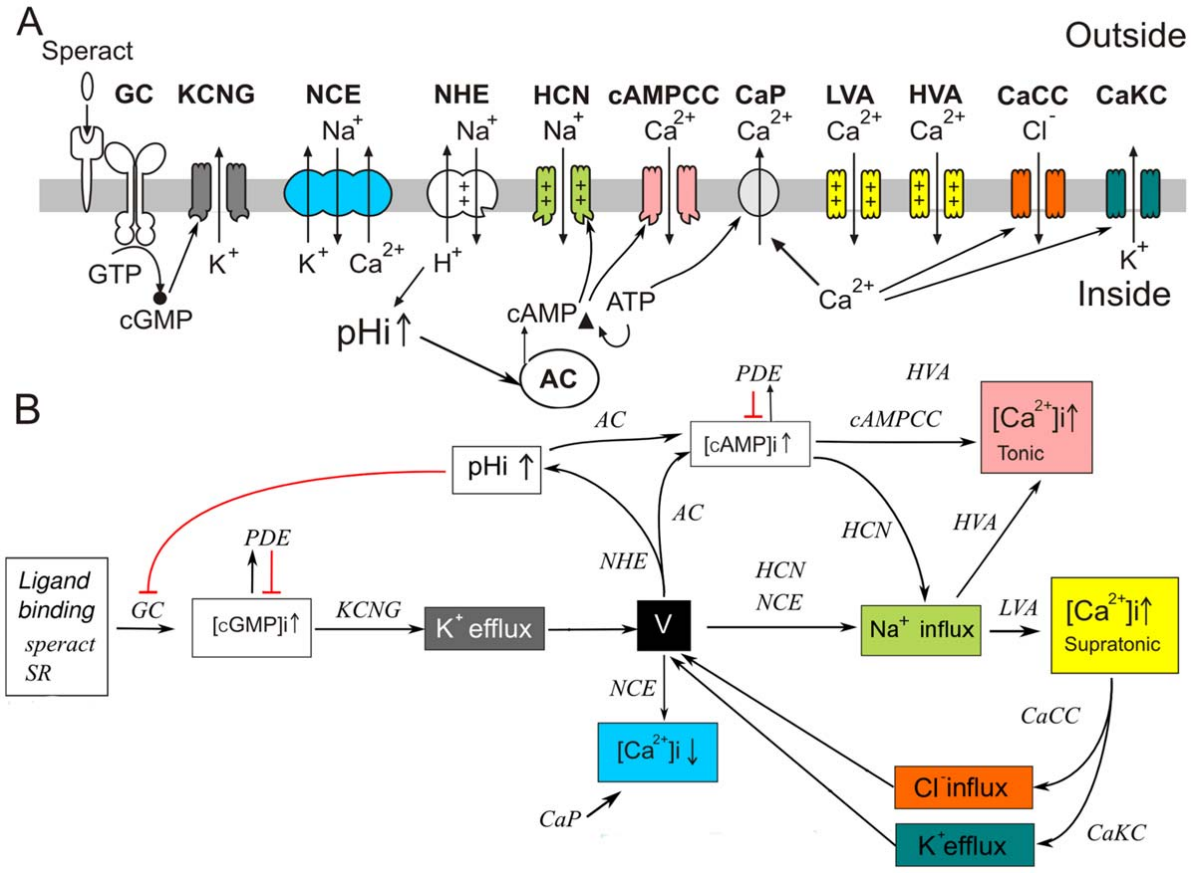
Signaling pathway triggered by speract in sea urchin sperm. A) Main components involved in the speract signaling pathway. The binding of speract with its receptor in the flagellar membrane triggers the cascade that produces changes in 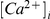 in two different forms: a sustained (tonic) increment and superimposed (supratonic) fluctuations. B) Events produced by the signaling pathway.

The dynamics of flagellar movement is triggered by a finely regulated signaling pathway depicted in Fig. 1. This pathway depends on the binding of speract to its receptor (SR) [2]–[4], [28], [29], which interacts with a membrane Guanylate Cyclase (GC) [30]–[33] that produces cGMP. The elevation of cGMP opens up a cGMP-regulated 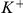 channel (KCNG) [6], [7], [12], [13], [34], [35], leading to a hyperpolarization of the membrane potential (V) [7], [8], [32]. This triggers the following processes:

1. Activation of a 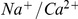 Exchanger (NCE) that decreases the flagellar 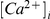 levels [32], [36], [37];
2. Activation of a 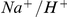 Exchanger (NHE) that increases intracellular pH (pHi) [8], [38];
3. Activation of a hyperpolarization-activated and cyclic nucleotide-gated channel (HCN) [32], [37], [39], [40];
4. Removal of inactivation of the high and low voltage-activated 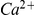 channels (HVA and LVA) [7], [9], [29], [41]–[43].

The pHi elevation decreases the GC activity and also activates a soluble adenylate cyclase (sAC) with the subsequent production of cAMP [32], [44], [45]. The latter stimulates a cAMP-dependent calcium channel (cAMPCC) and the previously activated HCN channel, which tends to repolarize the membrane potential [7], [13], [32], [37], [39]. Repolarization opens the above mentioned HVA and LVA channels causing a depolarization and an increment in 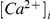 [7], [9], [13], [14], [29], [32], [41]. Finally, to restart the pathway a new hyperpolarization is needed. This could be achieved through a 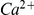-dependent 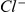 channel (CaCC) and a 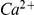-dependent 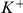 channel (CaKC) [29], [46], [47] which are opened when 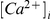 is high. Constant passive 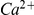 extrusion mechanisms, such as Calcium pumps (CaP) and NCE, maintain basal levels of 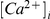 [32], [36], [37], [48]. The previous mechanism is then cyclically repeated to generate a train of 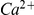 oscillations that produce a repetitive sequence of sperm turns [10], [11], [14], [41], [46].

### Network Dynamics

**Figure 2:**
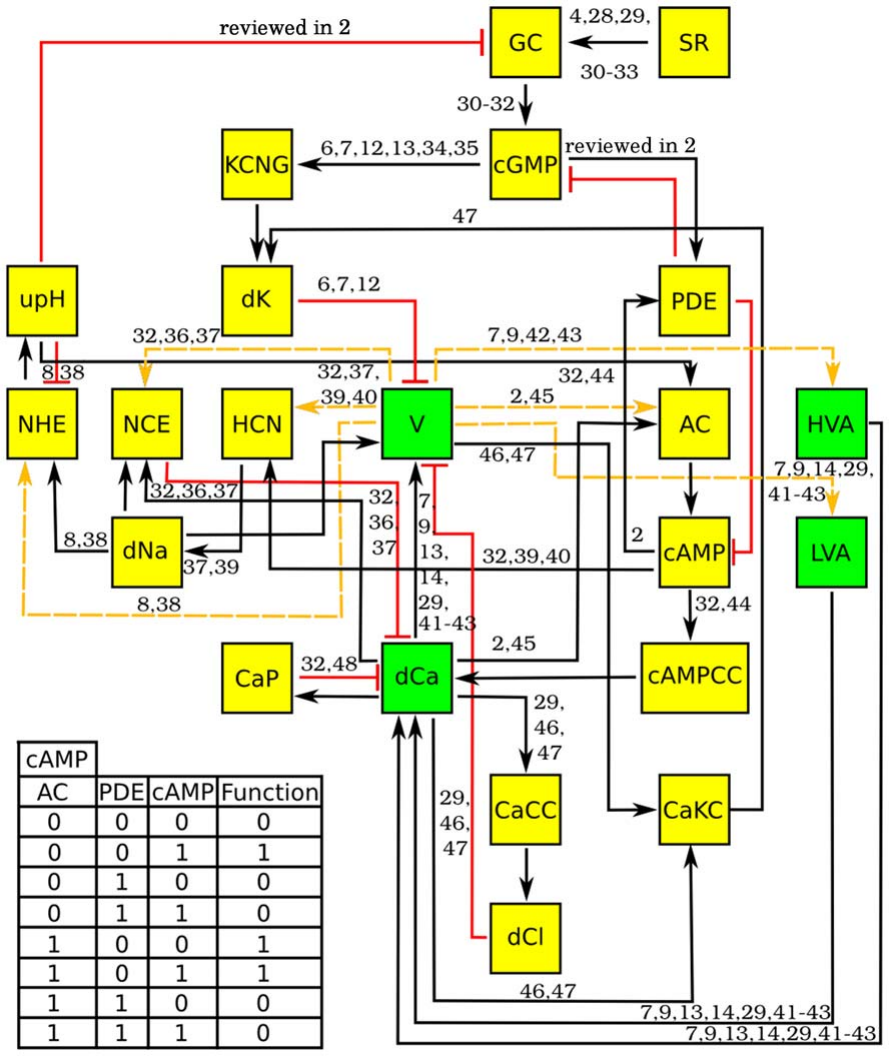
Speract-activated signaling logical network. Yellow and green boxes indicate binary and ternary nodes, respectively. Black arrows indicate activation, red lines inhibition and the yellow arrows can represent activation or inhibition depending on the value of the voltage node (v). Numbers over the arrows show references for the corresponding interaction. As an example, the regulatory function (or truth table) of the cAMP node is shown at the bottom left. The first 3 columns in this table contain all the possible activation states of the cAMP regulators (AC, PDE, cAMP); the fourth column shows the values for the function that correspond to each combination of the regulators.

Fig. 2 shows the logical signaling network corresponding to the speract-activated pathway described in the previous section. It consists of 22 nodes representing the principal components involved in the signaling cascade: ion channel activities, intracellular ion and molecular concentrations and the membrane potential, amongst others. To analyze the dynamics of the network, we implemented a discrete formulation that is a generalization of the Boolean approach and that has proven to be revealing for the gene regulation dynamics of many systems [49]–[53], as well as other cell signaling networks [54]. In this approach, the dynamical state of the network consists of a set of 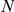 discrete variables 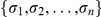, each representing the state of a node. For the particular network shown in Fig. 2, most of the variables take on two values, 0 and 1, depending on whether the corresponding element is absent or present, closed or open, inactive or active, etc. However, an accurate description of the dynamical processes in the network required four nodes to be represented by three-state variables: the membrane potential (hyperpolarized 0, resting 1, and depolarized 2); the low and high threshold voltage-gated 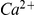 channels (inactive 0, closed 1, and open 2); and the intracellular calcium concentration 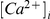 (basal 0, tonic 1 and supratonic 2). The state of each node 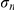 is determined by its set of regulators (which are some other nodes that also belong to the network). Let us denote as 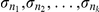 the 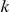 regulators of 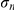. Then, at each time step the value of 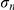 is given by
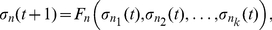
(1)where 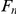 is a regulatory function constructed by taking into account the activating/inhibiting nature of the regulators. Each node has its own regulatory function. It is important to mention that there is not a general and systematic method to construct these regulatory functions. Instead, the construction of such functions is a craftwork for which it is necessary to know the specific nature of the regulatory interactions for each node. Sometimes all this information is not available and assumptions have to be made, especially regarding the concurrence of activating and inhibiting regulations (i.e. one has to decide which one is dominant). The inset in Fig. 2 is an example of the regulatory function for cAMP, which has a self-interaction. In this example, the node corresponding to the phosphodiesterase (PDE) is a strong inhibitor; if the PDE node is “on” at time 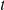, cAMP will be “off” at time 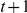, even if the other activator nodes are “on”. Other tables can become much more elaborate, such as the one for Ca which has 432 entries and involves 7 regulatory nodes, 3 of them with 3 states. For the construction of these regulatory functions, (which can be found at http://www.fis.unam.mx/research/seaurchin/discrete/), we have made use of all the biological knowledge, mainly of an electrophysiological nature, available to us in the literature and in our own laboratory.

Starting out the dynamics at time 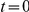 from any given initial state, 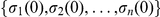, the network will traverse through a series of transitory states until it reaches a periodic pattern of activity called *attractor*. All the initial states that end up in a given attractor constitute the *basin of attraction* of that attractor. Several attractors may coexist for the same network, each with its own basin of attraction. For genetic networks, it has been shown that the dynamical attractors correspond to patterns of gene expression that determine the stable functional states of the cell (cell types or cell fates) [55]. In our case, the attractors of the signaling network will determine the stable calcium oscillations that drive sperm relocalization through 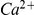-triggered flagellar curvature alterations. The period of the attractor is a qualitative representation of the time between calcium peaks.

We should point out that in the formalism we have adopted the values of the network nodes are updated synchronously. For an assessment of the limitations [56] involved in this assumption work is in progress by considering a semi-continuous model for the discrete dynamics here presented, following the piece-wise linear approach outlined by Glass [57]. In this formulation, nodes take continuous values and asynchrony is incorporated through a characteristic time scale for each node.

### Sea Urchin Sperm Experiments

To validate our network model we compared the outcoming dynamics with previously available experimental observations as well as with new experiments related to some of its predictions. In the new experiments here reported we looked into the alterations in the dynamics resulting from the elimination of elements in the network. For cases in which the ensuing dynamics showed noticeable modifications we determined the effect of experimentally blocking the corresponding eliminated elements. This was achieved by using the methodology of Wood et al [46] for uncaging speract (text S1), and testing the effect of several drugs known for their antagonistic effect on certain channels. We focused our attention on time related quantities such as the increase of the number of time-steps necessary to reach the first supratonic 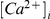 level and the times between successive 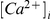 peaks, as well as changes in the intensity of 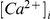 fluctuations (e.g. movies S1, S2 and S3). We looked into individual and population spermatozoa behaviors and undertook the appropriate statistical analyses. As shown in the Results section, in all the cases we obtained good agreement between the experimental determinations and the corresponding numerical simulations of the network model.

### Dynamical Regime

It is known that discrete networks can operate in three different dynamical regimes: ordered, critical and chaotic [58], [59]. These regimes are characterized by the manner in which perturbations are propagated across the network and how these perturbations change (or do not change) the network dynamical state. In order to define the perturbation cascade, let us consider two slightly different initial states which differ in the values of a small fraction of the nodes: 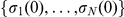 and 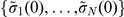. (For instance, the two states 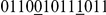 and 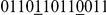 differ only in the values of the two underlined nodes.) Each of these initial states will generate a dynamical trajectory determined by Eq. (1). Let 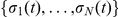 be the state of the network at time 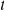 in the first trajectory, and 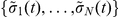 the corresponding state in the second trajectory. Then, we define 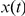 as the average number of nodes that are different in these two states at time 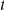, where the average is taken over many pairs of initial conditions. This quantity 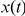 is a measure of the size of the perturbation avalanche at time 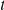 generated by the small difference in the two initial states. We hence have that the initial difference (perturbation) in the two states 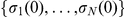 and 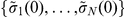 can affect the states of other nodes at the next time-step, which in turn affect the states of other nodes at the subsequent time-step, and so on, producing a perturbation cascade propagated in time by the network dynamics. In the ordered regime the network is said to be insensitive to perturbations because any perturbation cascade dies out over time (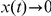 as 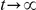), leaving the dynamical state of the network unchanged. On the contrary, in the chaotic regime, perturbations in the values of just a few nodes typically generate a perturbation cascade that progressively increases in time and propagates to the entire network altering its whole dynamical state (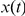 increases and reaches a finite nonzero value as 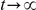). Finally, in the critical regime small perturbations typically neither increase nor decrease in time, but remain confined to a small subset of network elements (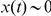).

**Figure 3:**
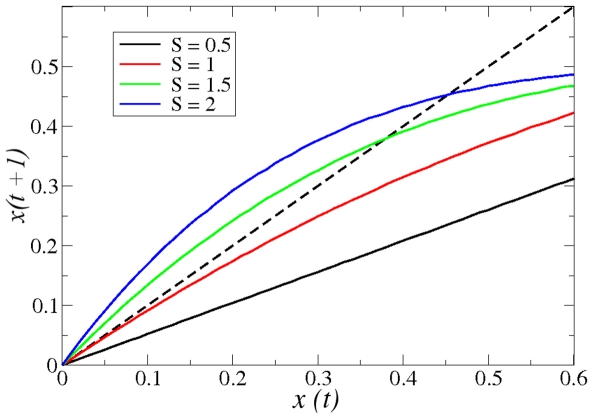
Derrida maps for random Boolean networks. This figure illustrates the Derrida map for networks operating in the three different regimes: Ordered (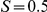, black), critical (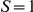, red), and chaotic (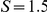, green and 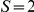, blue). Note that the Derrida map corresponding to the critical network (red curve) becomes tangent to the identity close to the origin.

To determine the dynamical regime in which the network operates, one can compute numerically the Derrida map 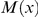, which relates the size of the perturbation avalanche at two consecutive time steps: 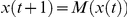. It is known that 
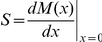
, i.e. the slope of this map at 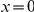, completely determines the dynamical regime: ordered if 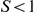, chaotic if 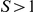 and critical if 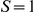 [24], [58], [59]. Fig. 3 illustrates this concept by showing the Derrida map for random Boolean networks operating in the three different regimes. Note that the Derrida curve corresponding to the critical regime becomes tangent to the identity close to the origin.

Networks that are critical with respect to the propagation of perturbations also exhibit other interesting properties. For instance, it has been shown that in critical networks the coexistence of robustness and adaptability, two central properties of living organisms, occurs with the highest probability [25]. They are also able to process, integrate and transfer information faster and more reliably than non-critical networks [60]. In general, dynamical criticality is an important property that has been observed in several complex systems, ranging from gene regulatory networks to neural activity in the brain [26], [27], [49].

## Results

### Network Time Evolution

**Figure 4:**
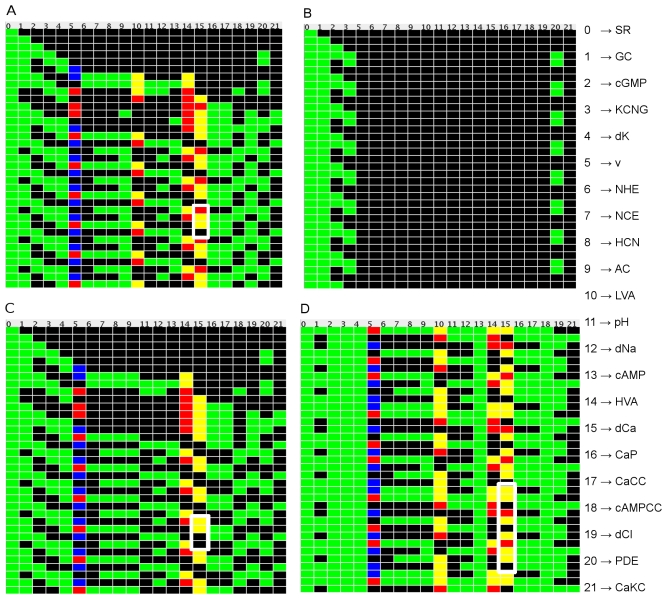
Dynamics of the signaling network. Activation pattern time-courses of the signaling network under different conditions. In each case, the nodes have been lined up horizontally, and are represented by rectangles colored according to their activation state: For the binary nodes black is “off” and green is “on”. For HVA and LVA Ca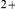 channels (nodes 10 and 14) black squares indicate inactive states, yellow are for closed states and red for open ones. For the membrane potential V (node 5), black squares indicate a resting potential, blue is hyperpolarization and red depolarization. For the Ca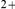 node (dCa) (node 15) we use yellow to indicate tonic elevation, red for a supratonic increment and black for the basal state. Starting out from an initial condition in which only the speract node is active, the dynamics unfold downwards, with each successive row representing the new dynamical state of the network at the next time step. A) Dynamics of the signaling network without deletions (all nodes present). The attractor has period 4 (for clarity, we indicated the period with a white frame for the calcium node, however, all nodes have the same periodicity). B) Agreement with experiment. Elimination of the K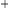 permeability node (dK) destroys the oscillations in practically all the nodes, particularly in the Ca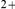 node. C) Agreement with experiment. Elimination of the LVA node suppresses the calcium supratonic states (red squares) without altering the periodicity of the attractor. D) Effect of the elimination of PDE node in the signalling network. When PDE node is eliminated, if we divide the number of supratonic states of calcium (red boxes) by the size of attractor (in this case is 11), the total of calcium (2/11) is less than the total of calcium in the entire network when the attractor is reached (1/4). This is according to the experiment when sperms are treated with IBMX, a blocker of phosphodiesterases. The correspondence between the numerical and alphabetical labels of the nodes is indicated at the right of the figure.

**Figure 5:**
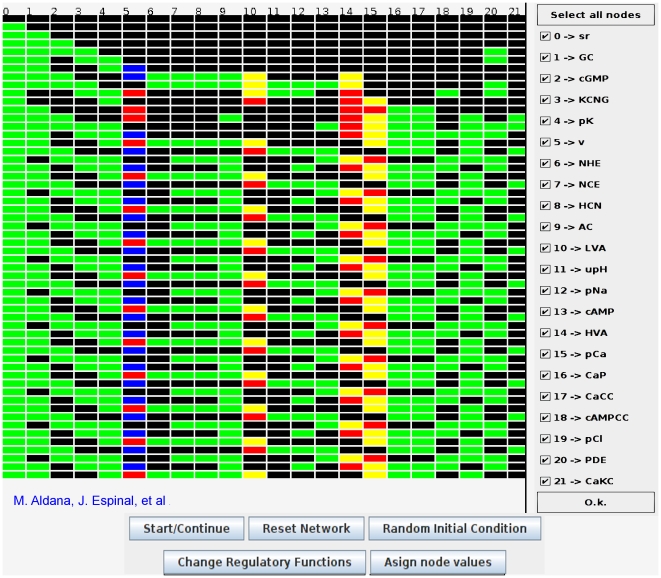
Snapshot of the Java applet that generates the dynamics of the sea urchin sperm signaling network. This applet can be found at http://www.fis.unam.mx/research/seaurchin/discrete/. The pattern is explained in the caption of Fig. 4. Be means of the applet buttons, it is possible to assign specific initial conditions or changes them randomly. It is also possible to explicitly visualize the regulatory functions of the network and modify them at will. Additionally, by unticking the boxes in the column to the right, it is possible to observe the effect in the pattern formation of deleting elements in the network. Further operational details can be found in the web page mentioned above.

The time evolution of the network is shown in Fig. 4a. In this figure each node is represented by a small square colored according to its value, and all the nodes are lined up horizontally. Thus, each row represents the dynamical state of the network at a given time (time unfolds downwards), whereas each column indicates the temporal evolution of a given node. The uppermost row is the initial condition with speract “on” (green square) and the rest of the nodes “off” (black squares). This corresponds to basal conditions with speract switched on. Note that after a small transient, a stage in which the configuration of the network is repeated after 4 time steps is attained for the whole system (the transient is the number of horizontal steps before the system reaches a recurrent pattern of squares). This period 4 behavior actually constitutes an attractor of the dynamics. Fig. 5 is a snapshot of the previously mentioned interactive applet that generates the signaling network evolution pattern for any initial condition, with the capabilities of retrieving and modifying any of the node regulatory functions. Node deletion and the observation of the induced changes in the patterns are also straightforward. The tool enables the corroboration of known experimental results as well as the prediction of previously unobserved behaviors testable by new experimental determinations.

### Attractors

**Figure 6:**
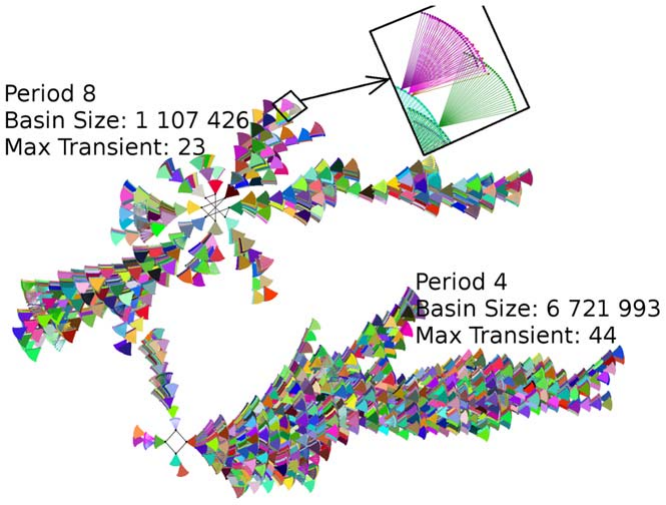
Graphical representation of the attractor landscape. The top right insert shows the fan-like structure where each dot represents a dynamical state of the network, and the lines represent discrete time steps. Two dots are connected if one is the successor of the other under the dynamics. The fan-like structures represent a set of different states that converge to a single state in one time-step. All the fan-like structures eventually converge towards the attractor, which is represented by the black dots connected by solid black lines. Only two active attractors with their attraction basins are shown: one of period 8 and the other one with period 4. The entire attractor landscape is shown in fig S1.

The network consists of 18 binary and 4 ternary nodes, giving a total of 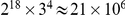 possible initial conditions for the dynamics. These possible initial conditions form the dynamical state space of the network, which in this case partitions into 11 attractors and their corresponding basins of attraction (Fig. 6 and Fig. S1). Six of these attractors have speract “off” and therefore are not biologically interesting, for they represent conditions in absence of speract. The other five attractors have speract “on” and represent active states of the signaling pathway. We will call these five attractors the active attractors. Four of the active attractors have period 4 and exhibit the same expression pattern for the calcium node. The fifth active attractor has period 8. This periodicity is shared by the calcium node. Thus, in the presence of speract our model predicts two different stable calcium oscillation patterns (Fig. 6). Since the period 8 basin of attraction is roughly one tenth of the period 4 basins, the probability of encountering it is low. However, our following results hold for all of the active attractors. It is important to mention that the six inactive attractors (the ones with speract “off”) are all point attractors (i.e. they all have period 1) and have very short transient times with respect to the transient times observed in the active attractors.

### Agreement with Previous Experiments

Experiments in this and the following sections were carried out following the protocol described in text S1. The elimination of nodes in the network gives the following results which are in accordance with previous experimental determinations:

1. In the absence of speract, for all initial conditions, after a small transient the system reaches basal conditions, hence confirming the role of speract in triggering the oscillations.
2. If the node corresponding to the potassium permeability (dK) is eliminated, 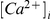 oscillations are suppressed as is shown in Fig. 4b. This behavior has been observed experimentally by working with an external medium with high 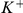 concentration [12], [29].
3. Nickel or nimodipine block T-type (low voltage activated) (LVA) 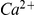 channels [29], [41], [43]. When added to the sperm external medium, the flagellar 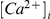 oscillations are inhibited. This behavior is reproduced by the network dynamics where under the elimination of the LVA node (Fig. 4c) the peaks of the supratonic response of 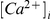, shown in red in Fig. 4a, disappear.
4. It is known experimentally that PDE inhibitors such as IBMX (3-isobuthyl-1-methylxanthine) eliminate speract-induced 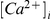 supratonic oscillations[29]. In our model the elimination of the PDE modifies the attractor by increasing the 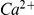 oscillation period from 4 to 11. The overall effect is that the ratio of supratonic events with respect to the attractor size (its periodicity) decreases from 1/4 to 2/11 (Fig. 4d). This behavior exhibits a trend that points in the direction of the experimental results.

### Predictions and Experimental Verifications

#### Role of 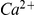-dependent 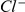 (CaCCs) and 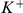 channels (CaKCs)

**Figure 7:**
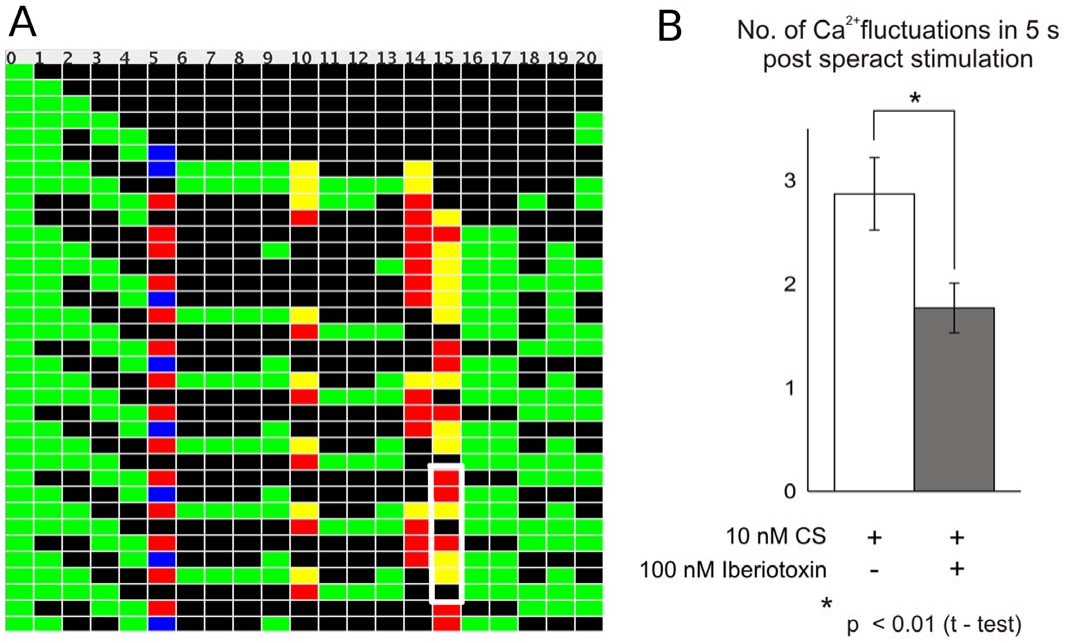
Effect of eliminating the CaKC node from the network. A) Typical realization of the network dynamics when the CaKC node is eliminated. In this case the periodicity of the attractor duplicates, and there is an increment in the supratonic states per period of the dCa node. B) This is again verified by experiments, showing that iberiotoxin, a specific blocker of the CaKC, reduces the number of speract-induced Ca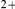 fluctuations that occur during the 5 sec immediately after stimulation. (movies S1, S2).

Experimental work has shown that niflumic acid (NFA) increases the amplitude and the time between successive peaks of the sea urchin 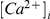 oscillations [29], [46]. This has been suggested to be due to a blockage of membrane channels that participate in the hyperpolarization subsequent to the first 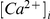 increase. It is known that NFA acts on CaCCs and CaKCs [47]. However, given its low specificity it might also target other elements of the signaling pathway. If we eliminate the CaCC node in our network simulations, the 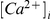 oscillation pattern is not altered. On the other hand, suppression of the CaKC modifies 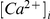 oscillations, producing an attractor with period 8 for all initial conditions with speract “on” and a higher density of supratonic events. This is illustrated in Fig. 7A, where it can be observed that there are more red squares per attractor period than in Fig. 4a. In this sense oscillations are more intense and less frequent [29], [46]. In order to carry out an experimental verification of this last result, spermatozoa were exposed to speract in the presence of Iberiotoxin, a potent and specific antagonist of CaKC activity. Iberiotoxin increased the period between successive speract-induced 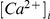 oscillations, in accordance with the model result of Fig. 7A, reducing the number of events that occur during the first five seconds after speract exposure (Fig. 7B). However, the amplitude of the 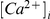 oscillations is lower than for the control group (movies S1 and S2). This could be due to a basal CaKC activity in the absence of speract. Blockage of this channel would depolarize, reducing the initial speract response. Further experiments are required to validate this explanation. It is worthwhile pointing out that, prompted by the model, to our knowledge for the first time, experiments were performed evidencing the participation of CaKC in the speract activated 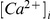 oscillation signaling pathway.

#### High voltage activated (HVA) 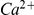 channel suppression

**Figure 8:**
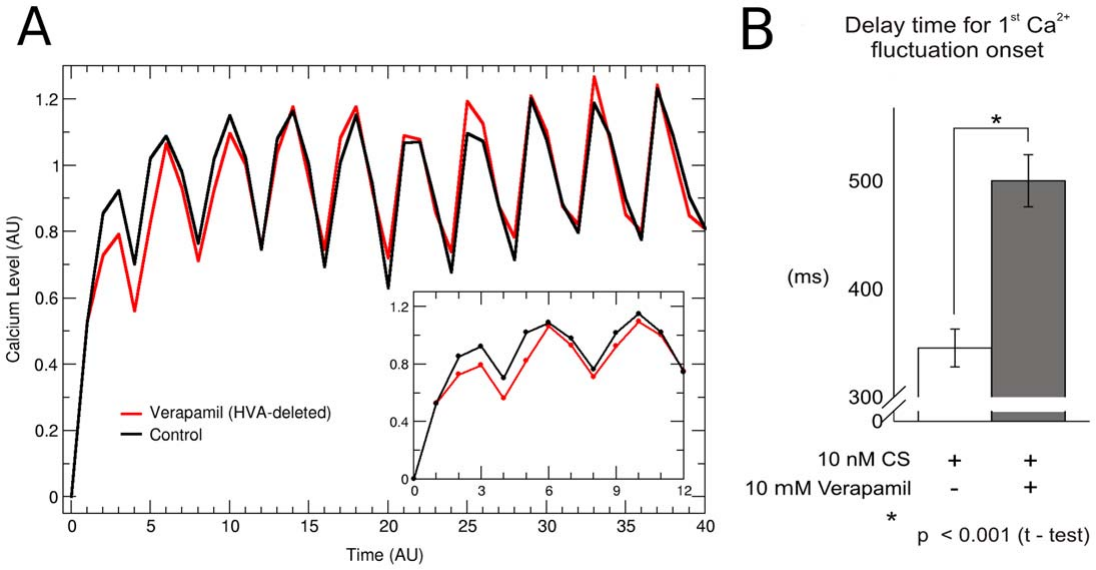
Effect of eliminating the HVA node. A) Temporal evolution of the average calcium level taken at each time step over 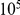 initial conditions with HVA (black curve) and without HVA (red curve) in arbitrary units. Note in the inset that when HVA is present the increase of the calcium level starts more rapidly than when HVA is deleted, i.e, in the initial time stages, the black curve preceds the behavior of the red curve. B) Experiments show that verapamil, an HVA inhibitor, prolongs the time between speract stimulation and the onset of the first Ca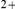 fluctuation (movie S2).

An important observations resulting from the network dynamics model is that suppression of the HVA node increases the time the calcium concentration takes to reach high intensity values after speract binding to its receptor. This is shown in the curves of Fig. 8A of the time evolution of the average of the calcium level taken over 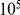 initial conditions. Note that in the transient behavior shown in the inset, the control curve precedes the HVA-deleted one. Prompted by this result we have carried out experiments adding 10 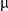
M of verapamil, an inhibitor of the HVA channel. Blockage of HVA produces a delay in the appearance of the first fluctuation (supratonic increase) in 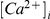 in the sperm flagellum, which verified the model prediction (Fig. 8B and movie S3).

### Criticality

**Figure 9:**
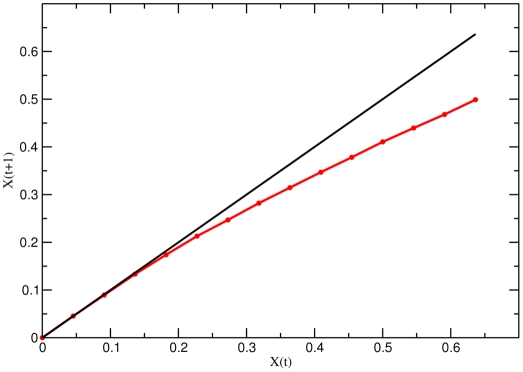
Critical dynamics in the calcium signaling network. Plot of the Derrida map 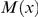 that relates the size of the perturbation avalanche at two consecutive time steps. The convergence of this map to a stationary value under successive iterations, determines the dynamical regime in which the network operates. The map shown here was computed numerically for the signaling network by relating an initial separation 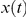 against separation 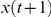 obtained after one step, averaged over all states initially separated by 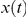. Notice that the slope of the curve near the origin is practically 1 in a sizeable neighborhood of the origin. This indicates that the signaling network operates in the critical regime.

Quite remarkably, the Derrida map 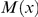 of the calcium network shows with high accuracy that this network operates in the critical regime. This is evident from Fig. 9, where it is shown that the slope at the origin is 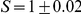. Systems operating close to a critical point have remarkable properties that would be very difficult to understand in the absence of criticality. As mentioned before, critical systems can process information faster and more reliably than non critical ones [60], [61]. Additionally, they can receive and integrate a wide range of external stimuli without saturating [26], or present collective responses in which all parts of the system are correlated [25]. In particular, a property of regulatory networks operating close to criticality relevant to the present study, is that they can be robust and evolvable at the same time, not only under transient environmental perturbations, but also under permanent internal reconfigurations (mutations) of the network [27]. This is important because sea urchin spermatozoa from different species encounter a wide variety of environmental conditions, therefore, the calcium signaling pathway must be robust enough to perform reliably under external perturbations. At the same time, it must be able to integrate the external signals and adapt to different environments. Such a delicate balance between robustness and adaptability is achieved with the highest probability in critical networks [27]. Therefore, the critical dynamics revealed in Fig. 9 is indicative of a highly selected mechanism optimizing efficiency in conjunction with flexibility in the flagellar beating.

## Discussion

Based on experimental evidence, we have built the network that describes the molecular processes involved in the signaling pathway of the 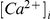 oscillations in the sea urchin spermatozoon that regulates its motility. By implementing discrete dynamics on the network we have been able to reproduce previous experimental results as well as to predict new behaviors. The predictions of the model have been corroborated with new experiments and have helped to clarify the role of different elements in the network. In particular we have suggested with our pharmacological experiments, the participation of the HVA channel in the determination of the transient time, between speract activation and the onset of calcium peaks (Fig. 8). We have experimentally verified this result by blocking the channel with verapamil, which leads to an increase in the delay time to the first calcium surge. Additionally, focusing on the calcium-dependent potassium channel in the network, we have uncovered its effect on the 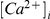 inter-peak time interval (Fig. 7). We have also verified this relation experimentally by exposing spermatozoa to Iberiotoxin. It is worth mentioning that the participation of this channel in the 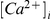 signaling pathway had been only suggested previously [46].

The model developed in this work also addresses general issues on how living machineries operate. Criticality in biological systems has been a matter of intense research [24], [26], [49]. This is the first time, to our knowledge, that critical dynamics have been encountered in the context of signaling pathways related to fertilization. With regard to this finding, it is worth mentioning that spermatozoa of *Lytechinus pictus* sea urchin present chemotaxis while those of *Strongylocentrotus purpuratus* do not (under tested experimental conditions), although they appear to share very similar 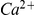 signaling pathways triggered by speract [10], [11]. Furthermore, operation under critical dynamics may contribute to explain behavioral diversity, since such a regime provides the flexibility for responding adequately to the different environments in which these species live. The system, while being robust in the sense that the main swimming mechanisms are preserved, has the capacity of adapting to the surroundings. It is important to stress that this network was constructed taking into consideration the experimental evidence regarding the stimulatory and inhibitory nature of the regulations. Criticality was never a relevant criterion in the construction of the network. However, the fact that the resulting network is dynamically critical with such a high accuracy supports the long-standing hypothesis that living systems operate close to condition of criticality at different levels of organization [25], [49], [59].

## Supporting Information

Figure S1 **Attractor landscape of the signalling network.** The six basins of attraction at the top correspond to states in which speract is “off”. In this case, all the attractors have period one (point attractors) and the transient times are relatively short. The five attraction basins at the bottom correspond to the active attractors in which speract is “on”. These are the biologically relevant attractors because they represent active states of the signalling pathway. Four of the active attractors have period 4 and one has period 8 (the period of each attractor is indicated by the bold number next to it). Note that in this case the transient times are much longer that the ones observed for the point attractors at the top (the fan-like structures form much longer “arms”). The period-4 attractors have almost the same activity pattern except for the ternary nodes HVA and LVA.
(TIFF)

Movie S1
**Iberiotoxin slowing down response.** Iberiotoxin reduces the number of speract
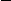
induced Ca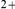 fluctuations that occur during the 5 s immediately after stimulation. Swimming Fluo-4 loaded *S. purpuratus* spermatozoa exposed to speract through the photo-release of Caged speract (10 nM) with a 200 ms UV flash alone (left panel) or in the presence of Iberiotoxin (100 nM, right panel). An optic liquid guide of 4000 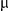
m internal diameter was used as light path between the UV lamp and the microscope. Pseudo-color scale representing maximum (red) and minimum (blue) relative fluo 4 fluorescence intensity. Five times slower: 30 frames s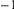, 40× objective.(AVI)

Movie S2
**Iberiotoxin slowing down response shown only for one sperm.** For clarity, one spermatozoon of each experimental condition described in Movie S1 is encircled (red); other spermatozoa were manually eliminated after speract photo
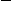
activation. Notice that the control presents twice the number of Ca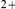 up surges.(AVI)

Movie S3
**Delay time for the onset of the first Ca** 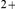 **fluctuation of** ***S. purpuratus*** **spermatozoa is increased by the presence of Verapamil.** Swimming Fluo
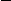
4 loaded *S. purpuratus* sperms were expose to speract through the photo
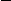
release of caged speract (10 nM) with a 200 ms UV flash alone (left panel) or in the presence of Verapamil (10 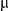
M, right panel). An optic fiber of 4000 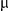
m internal diameter was used as light path between the UV lamp and the microscope. 20 times slower: 120 frames s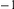, Scale bar = 25 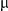
m, 40× objective. Yellow circles indicate spermatozoa at the beginning and progression of the first Ca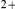 fluctuation until reach the averaged delay time of the whole population is reached.(AVI)

Text S1
**Experimental Protocol.** i)Materials and Methods. ii)Loading of 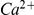 fluorescent indicator into spermatozoa. iii)Fluorescence imaging of swimming spermatozoa.(DOC)
