# Supplementary figures and images for: Discrete Dynamics Model for the Speract-Activated Ca2+ Signaling Network Relevant to Sperm Motility
Source: PLoS One. 2011 Aug 16;6(8):e22619. doi: 10.1371/journal.pone.0022619 (PMC3156703; doi:10.1371/journal.pone.0022619)

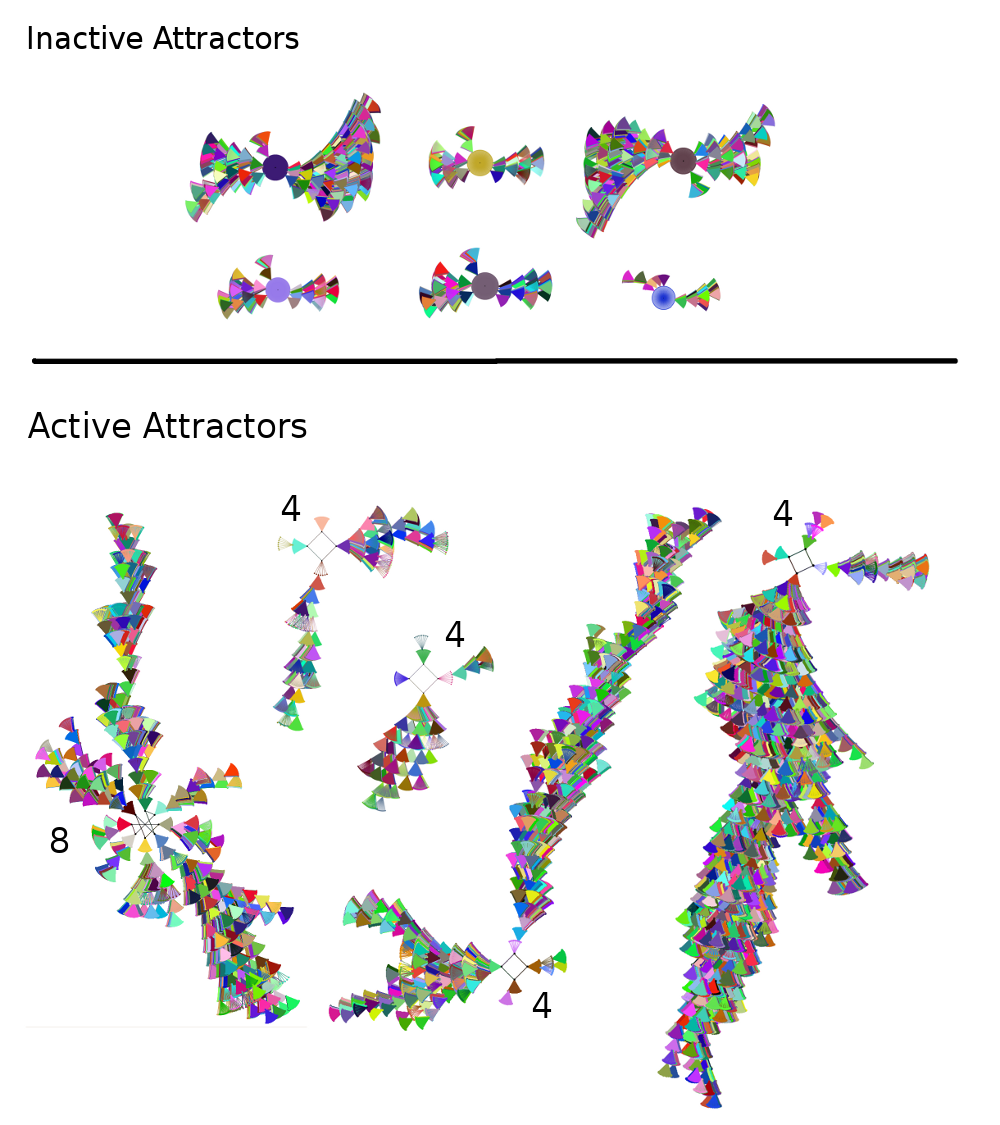

Supplement: Figure S1 — Attractor landscape of the signalling network. The six basins of attraction at the top correspond to states in which speract is “off”. In this case, all the attractors have period one (point attractors) and the transient times are relatively short. The five attraction basins at the bottom correspond to the active attractors in which speract is “on”. These are the biologically relevant attractors because they represent active states of the signalling pathway. Four of the active attractors have period 4 and one has period 8 (the period of each attractor is indicated by the bold number next to it). Note that in this case the transient times are much longer that the ones observed for the point attractors at the top (the fan-like structures form much longer “arms”). The period-4 attractors have almost the same activity pattern except for the ternary nodes HVA and LVA. (TIFF) [file pone.0022619.s001.tif]
