# Supplementary material for: Discrete Dynamics Model for the Speract-Activated Ca2+ Signaling Network Relevant to Sperm Motility
Source: PLoS One. 2011 Aug 16;6(8):e22619. doi: 10.1371/journal.pone.0022619 (PMC3156703; doi:10.1371/journal.pone.0022619)
Supplement: Text S1 — Experimental Protocol. i)Materials and Methods. ii)Loading of fluorescent indicator into spermatozoa. iii)Fluorescence imaging of swimming spermatozoa. (DOC) [file pone.0022619.s005.doc]

**Supplementary Material**

**Text S1.**

**Materials and methods:**

Spermatozoa were obtained ‘dry’ from *S. purpuratus* (Pamanes S. A. de C.V., Ensenada, Mexico) by intercoelomic injection of 0.5 M KCl and stored on ice until used within a day. Artificial seawater (ASW) was 950–1000 mOsm and contained (mM): 486 NaCl, 10 KCl, 10 CaCl_2_, 26 MgCl_2_, 30 MgSO_4_, 2.5 NaHCO_3_, 10 HEPES and 1 EDTA (pH 8.0). [Ser^5^; nitrobenzyl-Gly^6^] speract, referred to throughout this text as ‘caged *speract*’, was prepared as previously described (Tatsu, K., Nishigaki, T., Darszon, A., Yumoto, N., 2002, A caged sperm-activating peptide that has a photocleavable protecting group on the backbone amide, FEBS Lett. 525:20-24). Fluo-4-AM and pluronic F-127 were from Molecular Probes, Inc. (Eugene, OR, USA). PolyHEME (poly(2-hydroxyethyl methacrylate)) and other reagents, unless indicated, were from Sigma-Aldrich (Toluca, Edo de Mexico, Mexico).

**Loading of Ca^2+^ fluorescent indicator into spermatozoa:**

Dry *S. purpuratus* spermatozoa were suspended in 10 volumes of low Ca^2+^ ASW containing 0.2% wt/vol pluronic F-127 and 20 µM of Fluo-4 AM and incubated for 2 h at 14 ºC. Loaded *S. purpuratus* spermatozoa were then diluted with 100 volumes of low Ca^2+^ ASW, centrifuged for 10 min at 1,000 g and 4 ºC, and re-suspended in the original volume of low Ca^2+^ ASW. Spermatozoa were stored in the dark and on ice until use during the day.

**Fluorescence imaging of swimming spermatozoa:**

All coverslips were briefly immersed into a 0.05–0.1% (wt/vol) solution of polyHEME in ethanol, hot-air blow-dried to rapidly evaporate the solvent, and mounted on reusable chambers fitting a TC-202 Bipolar temperature controller (Medical Systems Corp.). The temperature plate was mounted on a microscope stage (Eclipse TE 300; Nikon) and maintained at a constant 15 ºC. Aliquots of labeled sperm were diluted in 2.5 x 10^5^ volumes of the appropriate ASW and transferred to an imaging chamber. Epifluorescence images were collected with a Nikon Plan Fluor 40× 1.3 NA objective using a Chroma filter set (ex, HQ470/40×; DC, 505DCXRU; em, HQ510LP) and recorded on a EMCCD Andor camera (DV887, Andor iXon). Fluorescence illumination was supplied by a Luxeon V Star Lambertian Cyan LED part # LXHL-LE5C (Lumileds Lighting LLC, San Jose, USA) attached to a custom-built stroboscopic control box. The LED was mounted into a FlashCube40 assembly with dichroic mirror M40-DC400 (Rapp Opto Electronic, Hamburg). LED output was synchronized to the Exposure Out signal of the EMCCD camera via the control box to produce a single 2 ms flash per individual exposure. The camera exposure time was set equivalent to flash duration (2 ms). Images were collected with Andor iQ 1.8 software (Andor Bioimaging, NC) at 120 fps in full-chip mode, binning = 4 x 4. Photolysis of caged speract was via a fiber optic coupled Xenon UV lamp (UVICO, Rapp Opto Electronic) filtered through a UV band-pass filter (270 – 400 nm) connected to the FlashCube40 and triggered by TTL unit (Andor Bioimaging, NC) connected to a Master 8 pulse generator (A.M.P.I., Jerusalem, Israel). The internal diameter of the optical fiber was 4 mm.
